# Effect of Multi-Mode Divergent Ultrasound Pretreatment on Hardness, Microstructure and Digestion of Acid-Induced Whey Protein Gels

**DOI:** 10.3390/foods13121926

**Published:** 2024-06-19

**Authors:** Yu Cheng, Xiaolong Shi, Georgina Benewaa Yeboah, Lihong Chen, Juan Wu

**Affiliations:** 1 School of Food and Biological Engineering, Jiangsu University, 301 Xuefu Road, Zhenjiang 212013, China; 2222118030@stmail.ujs.edu.cn (X.S.); ama.benewaa@yahoo.com (G.B.Y.); bush_hong@ujs.edu.cn (L.C.); wujuan@ujs.edu.cn (J.W.); 2 Institute of Food Physical Processing, Jiangsu University, 301 Xuefu Road, Zhenjiang 212013, China; 3 Faculty of Agricultural Engineering, Jiangsu University, Zhenjiang 212013, China; 4 School of Food and Health Sciences, Anglican University College of Technology, Nkoranza P.O. Box 78, Ghana

**Keywords:** ultrasound, frequency, whey protein, gel, acid induced, digestion

## Abstract

Whey protein was pretreated with multi-frequency ultrasound in mono-, dual-, and tri-frequency modes. The effect of multi-frequency ultrasound pretreatment on the hardness, chemical forces, and microstructure of acid-induced whey protein gel was investigated. Whey protein gels pretreated with dual- and tri-frequency ultrasound showed higher hardness (*p* < 0.05) and a denser network than mono-frequency ultrasound and control. Moreover, they had higher hydrophobic interaction and lower disulfide bonds than the control (*p* < 0.05). The effect of gel properties on digestion was evaluated using an in vitro static model. Whey protein gels pretreated with dual- and tri-frequency ultrasound had a higher proportion of large fragments in the oral boluses than in the control. Large fragments (>3.35 mm) in those samples were resistant to gastric digestion. Moreover, the tri-frequency ultrasound pretreatment of whey protein gel released the least free amino group during gastric digestion. In contrast, whey protein gel with the mono-frequency ultrasound pretreatment released the highest amount of free amino acid group during intestinal digestion. Findings from this study suggests that gel hardness and network density could modulate the digestion behaviors of protein gels.

## 1. Introduction

Protein gels are employed in various applications in food processing because proteins are considered safe and nutritious with excellent gelation properties [1,2,3,4]. Some foods, such as dairy desserts and jellies, are made by gelation. The gelation of proteins involves two main processes; protein denaturation/unfolding and aggregation into a self-supporting structure with a 3-dimensional network [1,3,5]. The gel structure is a suitable medium for delivering sensitive bioactive compounds or nutraceuticals. It could protect them from degradation during food processing and through harsh conditions within the gastrointestinal tract [1,4,6]. In this view, several attempts have been made to produce protein gels of different matrices that serve different purposes in the food industry [2,3,7,8,9,10].

In recent decades, ultrasound (US) technology has been used in various food applications as a green technology [11,12,13,14,15]. It is environmentally friendly, with improved food quality in extractions, fruit drying, freezing and thawing, fermentation, and protein modification. The US can exert these effects through acoustic cavitation. Cavitation is generated in the medium by the collapse and explosion of microbubbles coupled with physical and chemical effects [16,17,18]. These mechanical and chemical effects, together with the pressure fluctuations resulting from collapse and bubble explosions, lead to modifications in the protein structure [12,14,19,20,21]. Changes to the structure then alter the functionality and properties of the protein. US treatment has been demonstrated to reduce the size of protein molecules, enhance protein solubility, and improve protein emulsifying and gelling properties [14,20,22]. Furthermore, US has been shown to strengthen textural properties, gel microstructure, and the rheological properties of protein gels [23,24,25].

Although most ultrasonic pretreatments on proteins are conducted with a mono-frequency of 20 kHz, different US frequencies have been used to study their effect on food proteins. The effect of ultrasound frequencies between 20 and 52 kHz on soy protein extraction [26] was assessed. Interestingly, the mono-frequency of 20 kHz was not the optimal selection. Moreover, dual-frequency ultrasonic (DFU) and tri-frequency ultrasonic (TFU) pretreatments have resulted in higher efficiency in protein enzymatic hydrolysis than mono-frequency ultrasonic (MFU) pretreatment [20,27]. These results confirm that different US frequencies and modes affect food proteins differently [22].

Whey protein is one of the crucial proteins in food products. Improving the gel properties of whey protein is of interest to the food industry. Ultrasound, as an innovative technology in the food industry, has been explored for its application in enhancing the acid-induced gel properties of whey protein [28,29,30]. Although US has been shown to improve cold-induced gel’s properties [31,32,33], gastrointestinal breakdown after US treatment has not been studied in detail. Studying the breakdown pattern of these US-treated proteins is expedient, since the newly developed structure might affect digestion and nutrient release [5,34]. Moreover, previous research on acid-induced whey protein gel was conducted with mono-frequency ultrasound. Our previous work showed that DFU pretreatment produced heat-induced whey protein emulsion gels with better mechanical properties and digestion than MFU [35,36]. However, the effect of multi-frequency ultrasonic pretreatment on acid-induced whey protein gel’s properties and digestion remains unclear. Whey protein gels prepared with heat-induced and acid-induced methods might differ in amino acid profiles of digesta, functional properties [28,37], and potential applications.

Glucono-delta-lactone (GDL) is the most common acid used to prepare acid-induced protein gels. Therefore, this work aimed to study the effect of mono-, dual-, and tri-frequency ultrasound pretreatments on the characterization and digestion of GDL-induced whey protein gels.

## 2. Materials and Methods

### 2.1. Materials

Whey protein isolate (WPI) (protein content of 90.90%) was acquired from Hilmar Company (Hilmar, CA, USA). Pepsin, pancreatin, Glucono-delta-lactone (GDL), and 2,4,6-trinitrobenzene sulfonic acid (TNBS) were obtained from Sigma-Aldrich (St. Louis, MO, USA). All other reagents were analytically pure.

### 2.2. Ultrasound Pretreatment on Whey Protein

Whey protein solution (10% *w*/*v* dissolved in 10 mmol/L phosphate buffer pH 7.0) was pretreated with a tri-frequency ultrasound device (Meibo Biotechnology Co., Ltd., Zhenjiang, China) [38] at different ultrasound frequencies and times (MFU-20 kHz for 30 min, DFU-20/35 kHz for 15 min and TFU-20/35/50 kHz for 10 min) with pulse on and off times of 5 s and 2 s, respectively. Dual- and tri-frequency treatments were conducted in simultaneous mode. The ultrasonic power for each frequency was 100 W.

### 2.3. Whey Protein Gels (WPG) Preparation

Whey protein solutions were heated at 90 °C for 5 min and immediately cooled to 20 °C. GDL (0.50%, *w*/*w*) was added, and gels were allowed to set at 25 °C. The GDL-induced gels were stored at 4 °C overnight and were equilibrated at 20 °C for an hour before use.

### 2.4. Rheological Properties Analysis

A rheometer (DHR-1; TA Instruments, New Castle, DE, USA) was used to measure rheological changes during the gelation of the whey protein solutions. For viscosity, the whey protein solution was assessed using 40 mm plate geometry in flow-sweep mode at 25 °C with a shear rate from 0.1 to 100 s^−1^. For small deformation analysis, the whey protein solution was preheated at 90 °C for 5 min. GDL (0.50% *w*/*w*) was added and stirred for 2 min at 200 rpm on a magnetic mixer. The mixture was immediately loaded into the cup in a concentric cylinder and covered with silicone oil to prevent evaporation. The test was conducted using time oscillation mode for 3 h at 25 °C with a strain of 0.5% and a frequency of 1.0 Hz.

### 2.5. Turbidity Determination

The turbidity of the whey protein solution during gelation was determined at different times using absorbance at 500 nm with a UV-2800 Spectrometer (Unico Scientific Instrument Co., Ltd., Shanghai, China).

### 2.6. Gel Solubility

Gel solubility in different chemical solvents was carried out using the method described by Zhang et al. [39] with some modifications. Two grams of cold set whey protein gel was dissolved in 18 mL of four different solvents: (A) 50 mM sodium phosphate buffer pH 7.0, (B) solution A containing 8 M Urea, (C) solution A containing 0.5% sodium dodecyl sulfate (SDS), and (D) solution A containing 0.25% (*w*/*v*) 2-mercaptoethanol. The suspensions were then heated for 30 min at 80 °C and cooled to room temperature, followed by centrifuging at 5000× *g* for 15 min. The protein content in the supernatant was estimated using the Biuret method [40]. When subjected to 8 M Urea, SDS, and 2-mercaptoethanol, these solvents can disrupt the hydrogen bonds, hydrophobic interaction, and disulfide bonds between protein molecules in gels [41], resulting in the dissolution of the protein within the gel matrix. The solution’s dissolved protein content represented the gels’ main forces.

### 2.7. Gel Hardness

The hardness of the gel was measured by compression with a texture analyzer (TA-XT Plus, Stable Microsystems, Surrey, UK). With a gel strain of 30%, a 40 mm diameter aluminum cylindrical probe was applied at a pre-test, test, and post-test speed of 1 mm/s. Gel hardness was calculated as the average of at least six replicates.

### 2.8. Scan Electron Microscopy (SEM)

Whey protein gels (WPG) were cut into smaller sizes (2–5 mm) with a knife and soaked in glutaraldehyde (2.5% *v*/*v*) for 2 h. The samples were dehydrated in ethanol with serial concentrations of 50, 70, 80, 90, and 100% and then dried with a critical point drier Quorom k850 (Quorum Technologies Ltd., Ashford, Kent, UK). SEM (HITACHI SU8010, Hitachi, Ltd., Tokyo, Japan) was used to observe the microstructures of the gels after coating with gold.

### 2.9. Sodium Dodecyl Sulfate-Polyacrylamide Gel Electrophoresis (SDS-PAGE)

WPG was centrifuged at 2000× *g* for 10 min. The leached water and gel matrix (solid) were collected, respectively. Protein in the gel matrix was prepared using the same procedure as in Section 2.6 with the solvent containing 0.5% (*w*/*v*) SDS and 0.25% (*w*/*v*) 2-mercaptoethanol, and the supernatant was collected for gel electrophoresis. Proteins in the leached water and protein matrix were displayed with reducing SDS-PAGE [42] using WPI solution as a reference. The stacking gels and separation gels concentrations were 5% and (*v*/*v*) 12% (*v*/*v*), respectively.

### 2.10. Simulated Digestion of WPG

MFU, DFU, and TFU pretreated WP gel samples and control samples were digested using the INFOGEST method described by Minekus et al. (2014) [43] with modifications as outlined in our previous work [36]. WPG was fractured with a grinder to simulate oral chewing, followed by mixing with SSF in a 1:1 ratio without amylase at 37 °C for 2 min. Then, WPG boluses were mixed with SGF in a 1:1 ratio, and pH was adjusted to 3.0 with 1 mol/L hydrochloric acid. CaCl_2_ (final concentration of 0.15 mmol/L) was added, followed by Pepsin (2000 U/mL in the mixture). Gastric digestion was stopped by raising the pH to 7 with NaOH (1 mol/L) after 120 min. The digesta was passed through a sieve with pores of 1.4 mm. The filtrate was used for intestinal digestion. Gastric chyme was mixed with SIF in a 1:1 ratio, bile salts (concentration 10 mmol/L), and CaCl_2_ (0.6 mmol/L). Pancreatin (based on trypsin at 100 U/mL) was added. Digesta (0.2 mL) were withdrawn at 0, 2, 4, 6, 8, 10, 15, 20, 30, 45, 60, 90, and 120 min and frozen immediately in liquid nitrogen.

### 2.11. Particle Size Distribution of the Solid Digesta

Oral boluses and gastric digesta were passed through sieves with various pore sizes [36]. The contents on each sieve were washed for 5 min under gentle running water, collected, and dried at 100 °C for 24 h. The weights of the dried boluses were recorded.

### 2.12. Free Amino Group Determination

Free amino groups were determined at different time points during gastric and intestinal static digestion with the TNBS method described by Spellman et al. (2003) [44]. Absorbance was measured at 420 nm with UV-2800 Spectrometer (Unico Scientific Instrument Co., Ltd., Shanghai, China). The free amino acid content was determined using the standard curve of L-leucine.

### 2.13. Statistical Analysis

At least two independent experiments were conducted using fresh samples prepared at different times. ANOVA was used for analyses, and the significance difference was set at *p* < 0.05. Figures were representations of means and standard deviations.

## 3. Results

### 3.1. Effect of Ultrasonic Frequency Mode on the Viscosity of Whey Protein Solution

**Figure 1:**
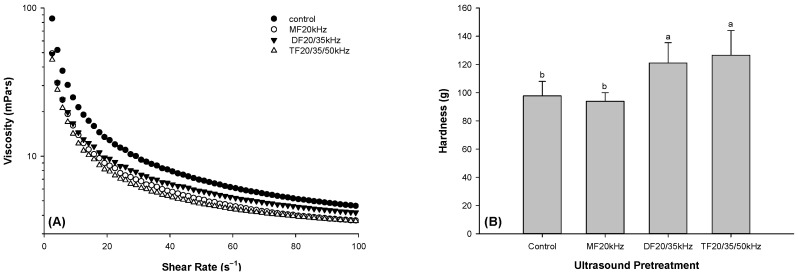
The viscosity of whey protein solution (**A**) and hardness of whey protein gel (**B**) pretreated with mono-, dual-, and tri-frequency (MF, DF, TF) ultrasound. Means with difference letters (a,b) are significantly different.

Ultrasound is known to decrease the protein viscosity by reducing the size of protein particles [45]. The reduction in particle size could be due to the unfolding and breakage of intermolecular hydrophobic bonds in the protein structure as an effect of acoustic cavitation. Our findings confirmed that multi-frequency US treatment decreased the viscosity of whey protein solutions compared to control, as shown in Figure 1A. Whey protein solutions with TFU treatment showed more thinning behavior than those with DFU pretreatment. However, with increasing shear, TFU and MFU showed a further decline in viscosity compared to DFU. This was unsurprising because DFUs of 20 and 35 kHz might lower the acoustic cavitation effect. The higher thinning effect in TFU could be due to its maximizing acoustic cavitation effect of the three frequencies on the whey solution. Although TFU treatment exhibited similar thinning behavior to MFU treatment, it could be more efficient because it utilizes less ultrasonic time.

### 3.2. Hardness of WPG

Figure 1B is a graphical representation of the hardness of GDL-induced WPG. It is evident from the graph that DFU and TFU pretreatment improved the hardness of gels. Compared with the control, the hardness of DFU- and TFU-pretreated WPG increased by 23.8% and 29.4%, respectively. Our results agreed with the existing literature [28] using MFU treatment. US improvement in hardness might be due to US-induced protein unfolding, increasing the availability of more protein bonds to strengthen the gel [46]. This was confirmed by previous studies on US-pretreated whey protein emulsion gel [36,47]. DFU-pretreated whey protein showed more protein unfolding than MFU, resulting in more rigid gels. The hardness and viscosity results of TFU-pretreated gel samples were consistent. Hardness was determined with solid whey protein gel samples, while viscosity was analyzed with whey protein solution at 25 °C. The lower viscosity suggested that the unfolding of the whey protein caused by the TFU pretreatment, may haveresulted in a more regular molecular orientation, decreasing the shearing resistance. The unfolding and regular molecular orientation could increase the interaction of protein molecules, resulting in a dense gel network and higher hardness.

### 3.3. Rheological Properties of WPG

**Figure 2:**
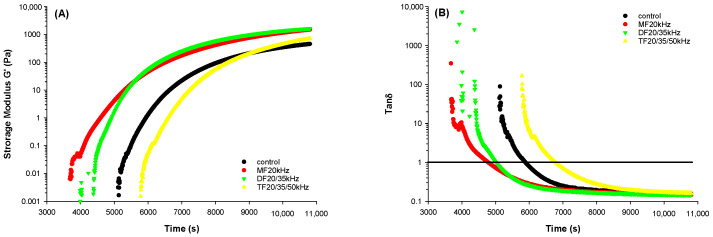
Storage modulus G’ (**A**) and tanδ (**B**) of whey protein solution pretreated with mono-, dual-, and tri-frequency (MF, DF, TF) ultrasound at different frequencies during time sweep.

Storage modulus (G’), which is a measure of gel rigidity, and tan delta (δ), which shows the viscoelastic property of gels, were studied (Figure 2). US treatments resulted in an improvement in the storage modulus of gels. This finding is in agreement with the existing literature [28,29], where the US caused significant improvement in the gel rigidity. DFU treatment gave a more rigid gel than MFU, which is in line with the findings of Cheng et al. (2019) [47], where DFU improved the rigidity (higher G’) of whey protein emulsion gels. This effect has been attributed to enhanced unwinding in the protein structure induced by the US, as it increases the availability of free proteins involved in gelation, bridging, and incorporation into strengthening the gel structure [36,47].

Nonetheless, WPG with TFU treatment had a lower G’ than DFU and MFU. The reason might be that WPG with TFU treatment exhibited a prolonged gelling time according to tanδ < 1 (Figure 2B). US is known to reduce the gelling time for gels [29]. However, our findings from this study contrasted this for TFU samples, as control samples gelled faster than TFU treatments (Figure 2B). This might be related to the different changes in protein structure. The gelling time affected the final G’. This was unsurprising because the time sweep interval was set at a limited time and could only represent the initial step of gel formation. Extending the time might lead to higher G’. The hardness result could be evident because G’ was positively related to the hardness of gels [28,47].

### 3.4. Protein Distribution in Whey Protein Gel

**Figure 3:**
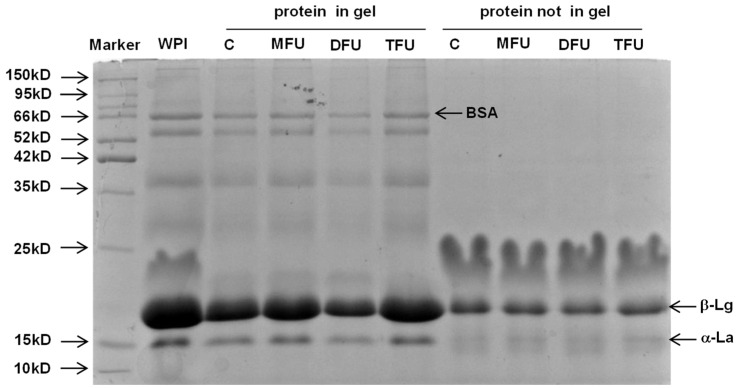
SDS-PAGE of protein distribution in whey protein gels with different ultrasound pretreatment (C: control without ultrasound, MFU: mono-frequency ultrasound, DFU: dual-frequency ultrasound, TFU: tri-frequency ultrasound).

To identify the proteins that were involved in gel formation, SDS-PAGE was carried out on US and control GDL-WPG after centrifugation (Figure 3). The results indicated that β-lactoglobulin was the main active protein in structuring the gel network regardless of treatment. Our results were consistent with previous studies that β-lactoglobulin was the main component responsible for gel networks [48,49]. Bovine serum albumin(BSA) and α-lactalbumin were present in the gel of all samples, indicating that they were responsible for strengthening and filling up the gel matrix. This finding demonstrated that the US did not affect the primary structure of proteins.

### 3.5. Turbidity and Chemical Forces

#### 3.5.1. Turbidity

**Figure 4:**
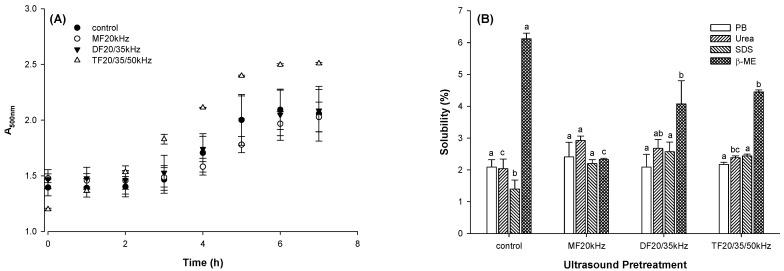
Turbidity of whey protein solution during gelation (**A**) and protein solubility of whey protein gel pretreated with mono-, dual-, and triple-frequency (MF, DF, TF) ultrasound in different chemical solvents (**B**) (the symbols a–c indicated the significant difference in US pretreatment).

The turbidity of the diluted protein solution was used to indicate the kinetics of protein aggregation during gel formation, as shown in Figure 4A. The kinetic model could fit a sinusoidal curve, indicating three stages of gel formation. From the onset of gel formation to 2 h, there were few changes in turbidity for control, MFU, and DFU. Beyond this, the turbidity of TFU increased with time. It is worth noting that the turbidity of DFU was similar to that of the control. Although TFU was less turbid from the onset of gel formation, this treatment produced the most turbid gel at the end of the 8 h. It suggested that TFU pretreatment led to more and larger fine protein aggregates. This was consistent with the results for gel hardness, for which TFU resulted in more rigid gels. This is because the protein aggregates from TFU samples could serve as sub-units and support the compact particulate gel network. The extended exponential growth stage of TFU-treated samples suggested they took longer to form a gel network, agreeing with the above rheological results that TFU-treated samples had longer gelation time.

#### 3.5.2. Chemical Forces

The chemical forces between protein molecules supported protein aggregates and gel networks. A solubility test was used to determine the possible forces stabilizing the gels in four different solvents (Figure 4B). Compared with the control, US generally increased gel solubility in urea and SDS (*p* < 0.05), indicating that US increased the number of hydrogen bonds and hydrophobic interactions in WPG. Meanwhile, the US decreased gel solubility in β-ME (*p* < 0.05), indicating that the US decreased the content of disulfide linkages. US treatment might have resulted in disulfide bond cleavages and sulphydryl group oxidation [28,50], reducing the number of disulfide bonds available for gel formation when compared to control. Disrupting disulfide bonds might improve molecular flexibility. It could increase the hydrogen bond and hydrophobic interactions between protein molecules. Hydrogen bonds and hydrophobic interactions are short-range forces. An increase in these chemical forces might lead to a shortened molecular distance. That could result in a compact gel network and strengthened gel texture.

For US-pretreated gels, MFU gels exhibited higher solubility in Urea than TFU gels (*p* < 0.05), showing that hydrogen bond content in MFU gels was higher than that in TFU gels. In contrast, disulfide content in TFU gels was higher than in MFU gels according to the solubility in β-ME (*p* < 0.05). MFU, DFU, and TFU gels had little difference in hydrophobic bonds supporting the gel structure. The reason might be that MFU, DFU, and TFU treatment resulted in little difference in the whey protein tertiary structure [35].

### 3.6. Microstructure of GDL-Induced Whey Protein Gel

**Figure 5:**
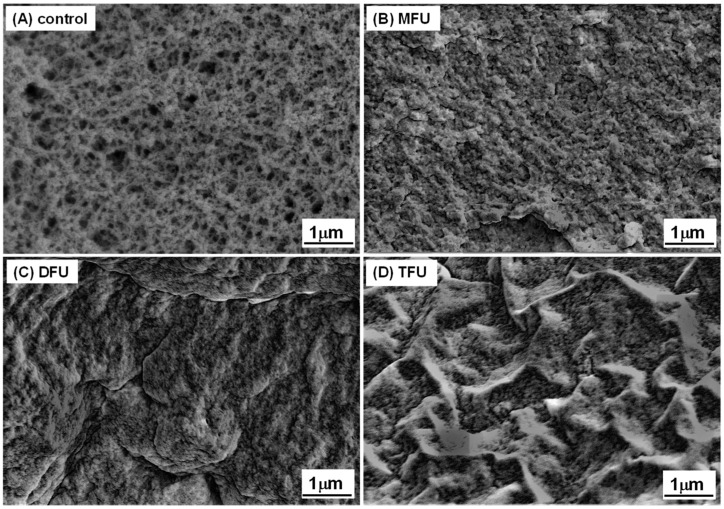
SEM images of whey protein gel prepared with different ultrasound pretreatments (control: without ultrasound, MFU: mono-frequency ultrasound, DFU: dual-frequency ultrasound, TFU: tri-frequency ultrasound).

SEM was used to study the gels’ microstructures, as shown in Figure 5. Our findings showed that the US could modify the microstructure of whey protein gel, as previously reported [50,51]. It was demonstrated that MFU, DFU, and TFU treatment produced compact gel microstructures and smaller pore sizes relative to the control, which exhibited larger pore sizes. The density of gels can impact their hardness, as a denser structure has greater resistance to deformation. Both DFU and TFU produced a more uniform homogenous gel network and dense particle cross-linking. This supports/corroborates the discussions on the chemical forces and gel hardness. TFU and DFU gave better results, with control samples yielding the softest gels. The increased compactness after US treatment could be due to unfolded protein structure [36,47], increased hydrogen bonds, and hydrophobic interactions in gel formation (Figure 4B). The higher short-range forces could increase intermolecular attraction by shortening the intermolecular distance, resulting in tiny pore sizes. Moreover, the US-induced microstructural changes might have resulted from the type of interactions involved in gel formation and different aggregation rates [52].

### 3.7. Digestion of GDL-Induced Whey Protein Gel

Our results showed that the US affected the microstructure of gels. Moreover, the structure of food plays an essential role in digestion, according to Dupont et al. (2018) [34]. With this idea, the simulated digestion of WPG samples was carried out for oral and gastrointestinal phases.

#### 3.7.1. Physical Digestion of WPG

**Figure 6:**
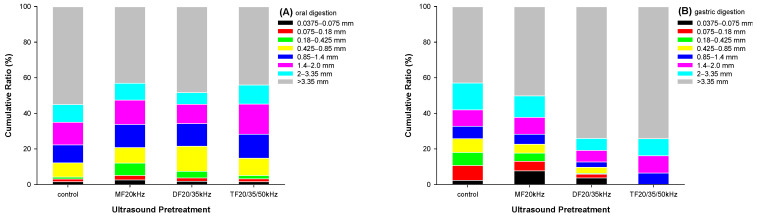
Particle size distributions in cumulative weight percentage of oral (**A**) and gastric (**B**) digesta from whey protein gel pretreated with mono-, dual-, and triple-frequency (MF, DF, TF) ultrasound.

The physical digestion of WPG during oral and gastric phases was investigated by sieving and estimated with a particle size distribution, as shown in Figure 6. After simulated mastication, US-pretreated WPG samples produced more larger fragments (>2.0 mm) and fewer small fragments (<0.425 mm) than the control (Figure 6A). This agrees with other research findings [53,54], where softer gels produced larger fragments than more rigid gels.

After 2 h of gastric digestion in the presence of pepsin, the weight percentage of DFU and TFU gastric fragments lower than 3.35 mm decreased by 48.1% and 52.5%, respectively, compared with oral fragments. By contrast, the weight percentage of the control sample gastric fragments higher than 3.35 mm decreased by 23.9%. This suggested that large boluses in the hard gel could resist pepsin digestion. The reason might be the difference in gel microstructure. The softer control sample had a loose structure in the gel network with a large pore size. It was easy for pepsin to diffuse into the gel network and interact with whey protein aggregates. It then degraded the large fragments in the boluses by attacking the protein aggregates’ interior and hydrolyzing the whey protein, breaking up the large boluses into smaller fragments.

On the contrary, the harder DFU and TFU WPG samples had a dense gel network with tiny pore sizes. The low porosity of the gel network might disturb pepsin diffusing into the interior of the protein aggregates, resulting in pepsin partitioning at the surface of protein aggregates [35]. This hindered the enzymatic degradation of the large boluses, leaving the gastric digesta in large sizes. On the other hand, US treatment could improve the digestion of whey protein [35,36], and fragments in small sizes decreased faster in US samples. Since the gastric chyme not exceeding 1.4 mm proceeded to the intestinal phase, little solid particles existed in the intestinal digesta. The existence of large fragments in gastric chyme could delay gastric emptying. This might affect the digestion characteristics of whey protein.

#### 3.7.2. Chemical Digestion of WPG

**Figure 7:**
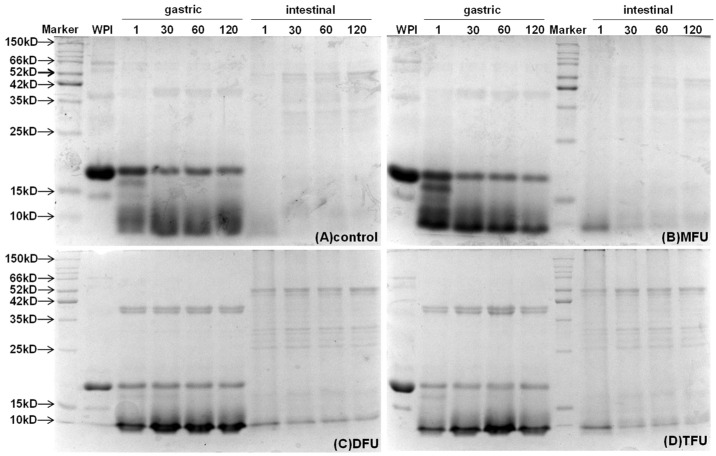
SDS-PAGE of ultrasound pretreated whey protein gels during gastric and intestinal digestion at different time points (1, 30, 60, 120 min) (control: without ultrasound, MFU: mono-frequency ultrasound, DFU: dual-frequency ultrasound, TFU: tri-frequency ultrasound).

SDS-PAGE was used to monitor the progress of WPG digestion at different times (Figure 7). Bands corresponding to β-lactoglobulin were persistent throughout gastric digestion (2 h), indicating the resistance of the gel structures to gastric digestion for all treatments. By contrast, the band of α-lactalbumin disappeared quickly when gastric digestion started. The bands of DFU and TFU were fainter than MFU with the progression of gastric digestion. DFU and TFU samples displayed light bands of β-lactoglobulin. This might be due to the resistance of gel structure and sensitive US-treated whey protein to pepsin hydrolysis. These results were in agreement with the results for particle size distribution. There were, however, no bands right from the onset of intestinal digestion, as shown in all samples. This pattern concurs with the literature that whey protein is a ‘fast protein’ hydrolyzed faster in the intestine and resistant to gastric digestion [53]. Thus, the US affected gastric digestion through its effect on gel structure but not intestinal digestion.

**Figure 8:**
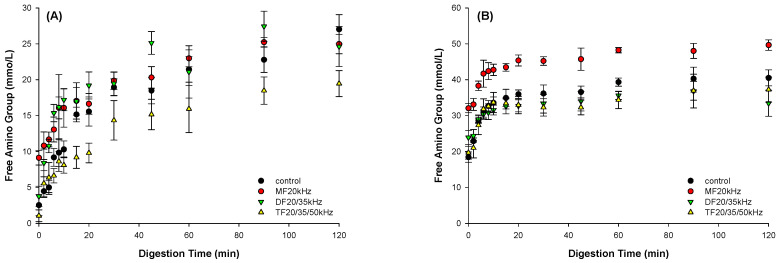
Effect of mono-, dual-, and triple-frequency (MF, DF, TF) ultrasound pretreatment on free amino group content in the liquid extracted from WPG gastric (**A**) and intestinal (**B**) digesta.

The hydrolysis of MFU and DFU released more amino groups than TFU throughout gastric digestion, as shown in Figure 8A, with the available amino group content ranging from 27.0 mmol/L (Control) to 19.4 mmol/L (TFU). This result indicated that the US influenced the degree of amino acid bioavailability; increasing the number of frequencies delayed gastric release of the amino group. This could be due to the inhomogeneous microstructure of the TFU gels, which minimized pepsin diffusion in the gel matrix. This is in line with Guo et al.’s results (2017) [55], where gels with more rigid structures showed limited protein hydrolysis and lower amino group release from the gel matrix. The structure of MFU best facilitated the release of amino groups compared to other treatments, including control during the intestinal phase (Figure 8B). This might be due to its coarse microstructure. The results show that MFU facilitated digestion with high amino group release compared to control, while TFU delayed gastric digestion.

## 4. Conclusions

This study showed that multi-frequency US could improve whey protein gels’ textural and rheological properties and delay their digestion. DFU and TFU treatments resulted in higher hardness and denser microstructure than MFU. Regarding digestion, the release of the amino group was higher in MFU-treated WPG due to its softer texture and coarse microstructure. The findings from this study showed that using the multi-frequency US as a food processing technique has implications for developing innovative protein gel products for nutrient delivery. It is recommended that the digestion pattern and kinetics of foods treated with US be investigated. As the static digestion model was used in this study, the physical fragment of WPG solids during the gastric phase was not considered. The dynamic digestion model will be used in further research to obtain more knowledge on the role of multi-frequency US.

## Data Availability

The original contributions presented in the study are included in the article, further inquiries can be directed to the corresponding author.

## References

[1] Cao Y.P., Mezzenga R. (2020). Design principles of food gels. Nat. Food.

[2] Hebishy E., Du H., Brito-Oliveira T.C., Pinho S.C., Miao S. (2023). Saltiness perception in gel-based food systems (gels and emulsion-filled gels). Crit. Rev. Food Sci. Nutr..

[3] Nascimento L.G.L., Odelli D., de Carvalho A.F., Martins E., Delaplace G., Peixoto P.P.D., Silva N.F.N., Casanova F. (2023). Combination of Milk and Plant Proteins to Develop Novel Food Systems: What Are the Limits?. Foods.

[4] Teimouri S., Kasapis S., Dokouhaki M. (2022). Diffusional characteristics of food protein-based materials as nutraceutical delivery systems: A review. Trends Food Sci. Technol..

[5] Siddiqui S.A., Alvi T., Biswas A., Shityakov S., Gusinskaia T., Lavrentev F., Dutta K., Khan M.K.I., Stephen J., Radhakrishnan M. (2023). Food gels: Principles, interaction mechanisms and its microstructure. Crit. Rev. Food Sci. Nutr..

[6] Abaee A., Mohammadian M., Jafari S.M. (2017). Whey and soy protein-based hydrogels and nano-hydrogels as bioactive delivery systems. Trends Food Sci. Technol..

[7] Cheng Q., Liu C., Zhao J., Guo F., Qin J., Wang Y. (2024). Hyaluronic acid modulates techno-functional and digestion properties of heat-induced ginkgo seed protein isolate gel. Food Biosci..

[8] Cheng Y., Ye A.Q., Singh H. (2024). Characterizations of emulsion gel formed with the mixture of whey and soy protein and its protein digestion under in vitro gastric conditions. Curr. Res. Food Sci..

[9] Grasberger K., Hammershoj M., Corredig M. (2024). Lupin protein-stabilized oil droplets contribute to structuring whey protein emulsion-filled gels. Food Res. Int..

[10] Zhang L.Y., Ge H.F., Zhao J., Liu C.Q., Wang Y.S. (2024). L-Theanine Improves the Gelation of Ginkgo Seed Proteins at Different pH Levels. Gels.

[11] Chen W.Q., Ding Y.H., Zhao Y.M., Ma H.L. (2023). Strategies to improve the emulsification properties of rice proteins as a promising source of plant-based emulsifiers: An updated mini-review. Food Biosci..

[12] Kang D.C., Zhang W.A., Lorenzo J.M., Chen X. (2021). Structural and functional modification of food proteins by high power ultrasound and its application in meat processing. Crit. Rev. Food Sci. Nutr..

[13] Pinton M.B., dos Santos B.A., Lorenzo J.P.M., Cichoski A.J.P., Boeira C.P., Campagnol P.C.B. (2021). Green technologies as a strategy to reduce NaCl and phosphate in meat products: An overview. Curr. Opin. Food Sci..

[14] Taha A., Mehany T., Pandiselvam R., Siddiqui S.A., Mir N.A., Malik M.A., Sujayasree O.J., Alamuru K.C., Khanashyam A.C., Casanova F. (2022). Sonoprocessing: Mechanisms and recent applications of power ultrasound in food. Crit. Rev. Food Sci. Nutr..

[15] Xu B.G., Tiliwa E.S., Yan W.Q., Azam S.M.R., Wei B.X., Zhou C.S., Ma H.L., Bhandari B. (2022). Recent development in high quality drying of fruits and vegetables assisted by ultrasound: A review. Food Res. Int..

[16] Bhangu S.K., Ashokkumar M. (2016). Theory of Sonochemistry. Top. Curr. Chem..

[17] Chandrapala J., Oliyer C., Kentish S., Ashokkumar M. (2012). Ultrasonics in food processing. Ultrason. Sonochem..

[18] Yusof N.S.M., Anandan S., Sivashanmugam P., Flores E.M.M., Ashokkumar M. (2022). A correlation between cavitation bubble temperature, sonoluminescence and interfacial chemistry—A minireview. Ultrason. Sonochem..

[19] Chen H.L., Xu B.G., Zhou C.S., Yagoub A.A., Cai Z., Yu X.J. (2022). Multi-frequency ultrasound-assisted dialysis modulates the self-assembly of alcohol-free zein-sodium caseinate to encapsulate curcumin and fabricate composite nanoparticles. Food Hydrocoll..

[20] Dabbour M., Jiang H., Mintah B.K., Wahia H., He R.H. (2021). Ultrasonic-assisted protein extraction from sunflower meal: Kinetic modeling, functional, and structural traits. Innov. Food Sci. Emerg. Technol..

[21] Qayum A., Rashid A., Liang Q.F., Wu Y., Cheng Y., Kang L.X., Liu Y.X., Zhou C.W., Hussain M., Ren X.F. (2023). Ultrasonic and homogenization: An overview of the preparation of an edible protein-polysaccharide complex emulsion. Compr. Rev. Food. Sci. Food Saf..

[22] Chen W.Q., Ma H.L., Wang Y.Y. (2022). Recent advances in modified food proteins by high intensity ultrasound for enhancing functionality: Potential mechanisms, combination with other methods, equipment innovations and future directions. Ultrason. Sonochem..

[23] Glover Z.J., Gregersen S.B., Wiking L., Hammershoj M., Simonsen A.C. (2022). Microstructural changes in acid milk gels due to temperature-controlled high-intensity ultrasound treatment: Quantification by analysis of super-resolution microscopy images. Int. J. Dairy Technol..

[24] Bangar S.P., Esua O.J., Sharma N., Thirumdas R. (2022). Ultrasound-assisted modification of gelation properties of proteins: A review. J. Texture Stud..

[25] Wang Q., Gu C., Wei R., Luan Y., Liu R., Ge Q., Yu H., Wu M. (2023). Enhanced gelling properties of myofibrillar protein by ultrasound-assisted thermal-induced gelation process: Give an insight into the mechanism. Ultrason. Sonochem..

[26] Ding Y.H., Ma H.L., Wang K., Azam S.M.R., Wang Y.Y., Zhou J., Qu W.J. (2021). Ultrasound frequency effect on soybean protein: Acoustic field simulation, extraction rate and structure. LWT-Food Sci. Technol..

[27] Xu B.G., Azam S.M.R., Feng M., Wu B.G., Yan W.Q., Zhou C.S., Ma H.L. (2021). Application of multi-frequency power ultrasound in selected food processing using large-scale reactors: A review. Ultrason. Sonochem..

[28] Shen X., Zhao C.H., Guo M.R. (2017). Effects of high intensity ultrasound on acid-induced gelation properties of whey protein gel. Ultrason. Sonochem..

[29] Gregersen S.B., Wiking L., Hammershoj M. (2019). Acceleration of acid gel formation by high intensity ultrasound is linked to whey protein denaturation and formation of functional milk fat globule-protein complexes. J. Food Eng..

[30] Shi R.J., Li T., Wang K.L., He Y.T., Fu R.X., Yu R., Zhao P.P., Oh K.C., Jiang Z.M., Hou J.C. (2021). Investigation of the consequences of ultrasound on the physicochemical, emulsification, and gelatinization characteristics of citric acid-treated whey protein isolate. J. Dairy Sci..

[31] Akdeniz V., Akalin A.S. (2019). New approach for yoghurt and ice cream production: High-intensity ultrasound. Trends Food Sci. Technol..

[32] Tomczynska-Mleko M., Nishinari K., Mleko S., Terpilowski K., Pérez-Huertas S. (2023). Cold gelation of whey protein isolate with sugars in an ultrasound environment. Food Hydrocoll..

[33] Zhao Y.L., Yan M., Xue S.Q., Zhang T.H., Shen X. (2022). Influence of ultrasound and enzymatic cross-linking on freeze-thaw stability and release properties of whey protein isolate hydrogel. J. Dairy Sci..

[34] Dupont D., Le Feunteun S., Marze S., Souchon I. (2018). Structuring food to control its disintegration in the gastrointestinal tract and optimize nutrient bioavailability. Innov. Food Sci. Emerg. Technol..

[35] Cheng Y., Yeboah G.B., Guo X.Y., Donkor P.O., Wu J. (2022). Gelling Characteristics of Emulsions Prepared with Modified Whey Protein by Multiple-Frequency Divergent Ultrasound at Different Ultrasonic Power and Frequency Mode. Polymers.

[36] Cheng Y., Donkor P.O., Yeboah G.B., Ayim I., Wu J., Ma H.L. (2021). Modulating the in vitro digestion of heat-set whey protein emulsion gels via gelling properties modification with sequential ultrasound pretreatment. LWT-Food Sci. Technol..

[37] Frydenberg R.P., Hammershoj M., Andersen U., Greve M.T., Wiking L. (2016). Protein denaturation of whey protein isolates (WPIs) induced by high intensity ultrasound during heat gelation. Food Chem..

[38] Mao C., Wu J., Zhang X., Ma F., Cheng Y. (2020). Improving the solubility and digestibility ofpotato protein with an online ultrasound-assisted pH shifting treatment at medium temperature. Foods.

[39] Zhang H., Wu J., Cheng Y. (2023). Mechanical properties, picrostructure, andin vitro digestion of transglutaminase-crosslinked whey protein and potato protein hydrolysate composite gels. Foods.

[40] Gornall A.G., Bardawill C.J., David M.M. (1949). Determination of serum proteins by means of the biuret reaction. J. Biol. Chem..

[41] Jiang J., Xiong Y.L. (2013). Extreme pH treatments enhance the structure-reinforcement role of soy protein isolate and its emulsions in pork myofibrillar protein gels in the presence of microbial transglutaminase. Meat Sci..

[42] You J., Liu C., Zhao J., Guo F., Wang Y. (2024). pH dominates the formation of ginkgo seed protein and whey protein composite gels. Food Biosci..

[43] Minekus M., Alminger M., Alvito P., Ballance S., Bohn T., Bourlieu C., Carrière F., Boutrou R., Corredig M., Dupont D. (2014). A standardised static in vitro digestion method suitable for food—An international consensus. Food Funct..

[44] Spellman D., McEvoy E., O’Cuinn G., FitzGerald R.J. (2003). Proteinase and exopeptidase hydrolysis of whey protein: Comparison of the TNBS, OPA and pH stat methods for quantification of degree of hydrolysis. Int. Dairy J..

[45] Meng Y.Y., Liang Z.Q., Zhang C.Y., Hao S.Q., Han H.Y., Du P., Li A.L., Shao H., Li C., Liu L.B. (2021). Ultrasonic modification of whey protein isolate: Implications for the structural and functional properties. LWT-Food Sci. Technol..

[46] Ramírez-Suárez J.C., Xiong Y.L. (2003). Effect of transglutaminase-induced cross-linking on gelation of myofibrillar/soy protein mixtures. Meat Sci..

[47] Cheng Y., Donkor P.O., Ren X.F., Wu J., Agyemang K., Ayim I., Ma H.L. (2019). Effect of ultrasound pretreatment with mono-frequency and simultaneous dual frequency on the mechanical properties and microstructure of whey protein emulsion gels. Food Hydrocoll..

[48] Rabiey L., Britten M. (2009). Effect of protein composition on the rheological properties of acid-induced whey protein gels. Food Hydrocoll..

[49] de Faria J.T., Minim V.P.R., Minim L.A. (2013). Evaluating the effect of protein composition on gelation and viscoelastic characteristics of acid-induced whey protein gels. Food Hydrocoll..

[50] Ma W., Wang J., Xu X., Qin L., Wu C., Du M. (2019). Ultrasound treatment improved the physicochemical characteristics of cod protein and enhanced the stability of oil-in-water emulsion. Food Res. Int..

[51] Ahmadi Z., Razavi S.M.A., Varidi M. (2017). Sequential ultrasound and transglutaminase treatments improve functional, rheological, and textural properties of whey protein concentrate. Innov. Food Sci. Emerg. Technol..

[52] Kharlamova A., Nicolai T., Chassenieux C. (2019). Heat-induced gelation of mixtures of casein micelles with whey protein aggregates. Food Hydrocoll..

[53] Guo Q., Ye A., Lad M., Dalgleish D., Singh H. (2014). Effect of gel structure on the gastric digestion of whey protein emulsion gels. Soft Matter.

[54] Luo N., Ye A., Wolber F.M., Singh H. (2020). In-mouth breakdown behaviour and sensory perception of emulsion gels containing active or inactive filler particles loaded with capsaicinoids. Food Hydrocoll..

[55] Guo Q., Bellissimo N., Rousseau D. (2017). Role of gel structure in controlling in vitro intestinal lipid digestion in whey protein emulsion gels. Food Hydrocoll..

